# MALDI-TOF MS, following separation gel coagulation tubes and short-term solid phase culture effectively detects pathogenic microbes from positive blood cultures

**DOI:** 10.1515/med-2025-1302

**Published:** 2026-04-17

**Authors:** Shujuan Chen, Kangying Wang, Haijian Tu

**Affiliations:** Department of Clinical Laboratory, The Affiliated Hospital (Group) of Putian University, Putian, Fujian, China

**Keywords:** matrix-assisted laser desorption ionization time-of-flight mass spectrometry, blood culture, Gram-negative bacteria, separation gel coagulation tube, direct identification

## Abstract

**Objectives:**

This study explored the viability of combining separation gel coagulation tubes with a short-term solid-phase culture method and matrix-assisted laser desorption/ionization-time of flight mass spectrometry (MALDI-TOF MS) for the detection of pathogens in positive blood culture bottles.

**Methods:**

Positive blood culture bottles (186) were collected from the Affiliated Hospital of Putian University. The blood culture bottles were processed using separation gel coagulation tubes to isolate plasma components. After centrifugation, the solid phase containing microbial cells was subjected to a 4–6 h short-term solid-phase culture. Bacterial identification was subsequently done using MALDI-TOF MS.

**Results:**

The genus and species identification coincidence rates for 107 Gram-negative bacteria were 90.7 % (97/107) and 86.0 % (92/107), respectively, and 73.7 % (56/76) and 72.4 % (55/76), respectively, for 76 Gram-positive bacteria. For the 37 isolates that were either identified only to the genus level or not identified, the coincidence rates of MALDI-TOF MS after 4 and 6 h of short-term solid-phase culture were 32.4 % (12/37) and 86.5 % (32/37), respectively.

**Conclusions:**

The combination of separation gel coagulation tubes, short-term solid-phase culture, and MALDI-TOF MS is fast and effective for identifying positive blood culture bottles in routine testing in clinical microbiology laboratories. Gram-negative bacteria are accurately and quickly identified compared to Gram-positive bacteria.

## Introduction

Bloodstream infections (BSI) have recently increased, resulting in high mortality rates and longer hospital stays [1]. According to a six-year retrospective study in the United States, the bacteremia incidence and death rate among hospitalized patients were 5.9 and 15.6 %, respectively [2]. In the United Kingdom, sepsis causes over 100,000 hospital admissions and 37,000 deaths annually [3]. Bloodstream infection diagnosis requires multiple positive blood culture results from different body parts. This confirms the source of the patient’s disease infection, aiding in the timely and targeted administration of antibiotics to reduce treatment costs, shorten hospitalization time, and improve the patient’s prognosis.

Traditionally, blood culture (BC) is regarded as the conventional standard for diagnosing bloodstream infections [4]. While BC is precise, results usually take over 24 h, and sample analysis might take even longer, particularly with slow-growing fungi or bacteria [5]. Prompt antibiotic commencement for patients with bloodstream infections is crucial to curb rising antimicrobial resistance, necessitating rapid and precise diagnosis. Besides, according to previous research, antibiotic treatment within 3 h of sepsis diagnosis can reduce the mortality rate by 14 % compared to patients who begin treatment after 3 h [6]. This phenomenon restricts the efficacy of BC, which identifies infections within at least 48 h [7], 8].

Matrix-assisted laser desorption ionization–time of flight mass spectrometry (MALDI-TOF MS) is a technique modified and used by Anhalt and Fenselau for rapid identification [9]. The approach is an alternative to other methods, including blood culture and biochemical identification tools, as the first line of pathogen identification. Due to the high resolution, sensitivity, and accuracy of MALDI-TOF MS [10], rapid and accurate diagnosis is most likely, thus enabling prompt treatment for improved clinical management. In addition, MALDI-TOF MS has effective detection capabilities for both Gram-negative and Gram-positive bacteria, and resolves constraints in the biochemical identification of anaerobic, fastidious bacteria, yeasts, and other difficult-to-culture and identify microorganisms [11]. As a result, the technique is becoming more relevant in clinical microbial diagnosis and is likely to supplant conventional biochemical methods [10], 12]. However, one of the limitations of MALDI-TOF is that it needs pure culture with adequate biomass, and low biomass or mixed culture specimens lead to unreliable results [8]. Consequently, an approach integrating multiple approaches is necessary to improve reliability and accuracy. The present study aimed to assess the efficacy of the separation gel coagulation tube method combined with the short-term solid-phase culture method and MALDI-TOF MS for identifying positive blood culture bottles while also comparing these results with those from conventional culture to assess the viability of the rapid approach.

## Materials and methods

### Study design and participants

The study was approved by the Ethical Committee of the Affiliated Hospital of Putian University under the approval number 2022051-XZ0. In total, 186 positive blood culture specimens from outpatients and inpatient departments of the Affiliated Hospital of Putian University were collected from January to December 2021. Positive blood culture samples validated using the Gram stain microscopy were included. Samples that had no bacteria or those with two or more bacteria types upon confirmation through microscopy were excluded. In cases where multiple sets of blood cultures tested positive simultaneously, only the first bottle with a positive result was analyzed. In contrast, all other bottles were disregarded to avoid duplication and ensure data accuracy.

### Instruments and reagents

The equipment used included BacT-ALERT 3D 120 blood culture system (BioMérieux, Canada) and Vitek MS mass spectrometer (BioMérieux, Canada). The reagents included formic acid ≥95 % purity (Sigma-Aldrich, St. Louis, Missouri, USA), absolute ethanol, ≥99.8 % purity (Thermofisher Scientific, Waltham, MA, USA), and α-cyano-4-hydroxycinnamic acid (HCCA) matrix was (Bruker Daltonics, Bremen, Germany). The metal target plate was purchased from Zhengzhou Antu Bioengineering Co., Zhengzhou, China. The chocolate agar, Columbia blood agar, and MacConkey agar plates were purchased from BioMérieux, Canada. The Schaedler agar was purchased from Nanjing Duly Biotech Co., Ltd, Jiangsu, China. Acetonitrile was purchased from Sigma Reagent Co., Burlington, Massachusetts, United States of America. The separation gel coagulation tube was purchased from Suzhou BD Medical Devices Co., Ltd, Suzhou, China.

### Quality control

*Klebsiella aerogenes* (ATCC 13048), *Candida glabrata* (ATCC 2590), and *Escherichia coli* (ATCC 8739) were used for quality controls.

### Conventional identification method (CID)

The blood culture bottles were analyzed after the bacT/ALERT^®^ 3D system flagged them as positive. Analysis of bacterial samples was done using Gram staining, then by subculture on a suitable solid agar medium for aerobic samples (MacConkey, Columbia or Chocolate agar), while anaerobic samples were inoculated onto the Schaedler agar and kept in an incubator HERAcell^®^ 240 i (Thermo Scientific, Langenselbold, Germany) at 35 °C for 18–24 h. The Schaedler agars were incubated in an anaerobic jar (Oxoid Ltd., United Kingdom) containing GasPak (Thermo Fisher Scientific, USA). Colonies cultured on the plate overnight were mounted on a glass slide and later prepared for assessment using VITEK MS^®^ System software (version 3.0, bioMérieux), following the manufacturer’s guidelines. Direct mass spectrometry detection was avoided where multiple bacteria were identified. The confidence values between 60 and 99.9 meant a decision on the correct identification was reached, as shown by the manufacturer. This system was referred to as CID.

### MALDI-TOF MS direct identification following the separation gel coagulation tube method

Approximately 5 mL of positive blood culture medium was drawn into the separation gel coagulation tube and centrifuged at 1,500 g for 10 min. The supernatant was discarded, leaving the gray-white bacterial enrichment on the surface of the separation gel. Next, 500 μL of sterile water was added to the bacterial enrichment on the surface of the separation gel and transferred to a 1.5 mL Eppendorf tube. The sample was centrifuged at 16,000 g for 2 min, the supernatant was discarded, and the specimen was washed twice using 500 μL of absolute ethanol. The bacterial enrichment was divided into two halves. After the first enrichment, the supernatant was discarded, 500 μL of 75 % ethanol was added and well mixed, centrifuged at 16,000 g for 2 min, and the supernatant (ethanol) was finally discarded. The sample was then centrifuged again at 16,000 g for 2 min. Later, the lid was opened and dried to remove the residual ethanol. Next, 20 μL of 70 % formic acid (depending on the number of bacteria) was added to the centrifuge tube and mixed, an equal amount of acetonitrile was added, and centrifuged at 16,200 g for 2 min. Approximately 1 μL of the supernatant was picked, smeared on the target plate, dried, and 1 μL of the a-cyano-4-hydroxycinnamic acid matrix solution was added dropwise. The sample was dried and loaded into the mass spectrometer for identification.

### Short-term culture

After short-term solid-phase culture for positive blood culture specimens that were only identified to the genus level or not identified, the identification method of short-term culture on solid culture medium can be combined. The second enrichment was transferred to a Columbia blood agar plate. A mixture of bacterial solution from the positive blood culture bottle was mixed. The sample was cultured in a 35 °C incubator with 5 % CO_2_ for 4–6 h to form a biofilm layer. Mass spectrometry identification was then directly conducted using VITEK MS, and the results were recorded. Formic acid pretreatment was applied during direct bacterial film processing for MALDI-TOF MS sample preparation to enhance protein extraction and improve spectral quality. The 4 and 6 h bacterial films were taken, and the target plate was directly smeared for MALDI-TOF MS identification.

### Statistical analysis

Data management was done using Microsoft Office Excel 2024 (Microsoft Corp., Redmond, WA) or Microsoft Office Access 2024 (Microsoft Corp.). Data analysis was done using GraphPad Prism. Bacterial identification obtained using the modified rapid methods was compared with that from the conventional methods. The results of bacterial identification were classified as correct at the species or genus level, with the calculation of unreliable identification and success rates. The statistical significance was set at p<0.05.

### Ethics statement

The study was done following the 1964 Helsinki Declaration and the relevant approval was done by the Ethics Committee of the Affiliated Hospital of Putian University under the approval number 2022051-XZ0.

### Informed consent

The participants were included in the study after a written informed consent.

## Results

### Pathogen distribution and composition

**Figure 1:**
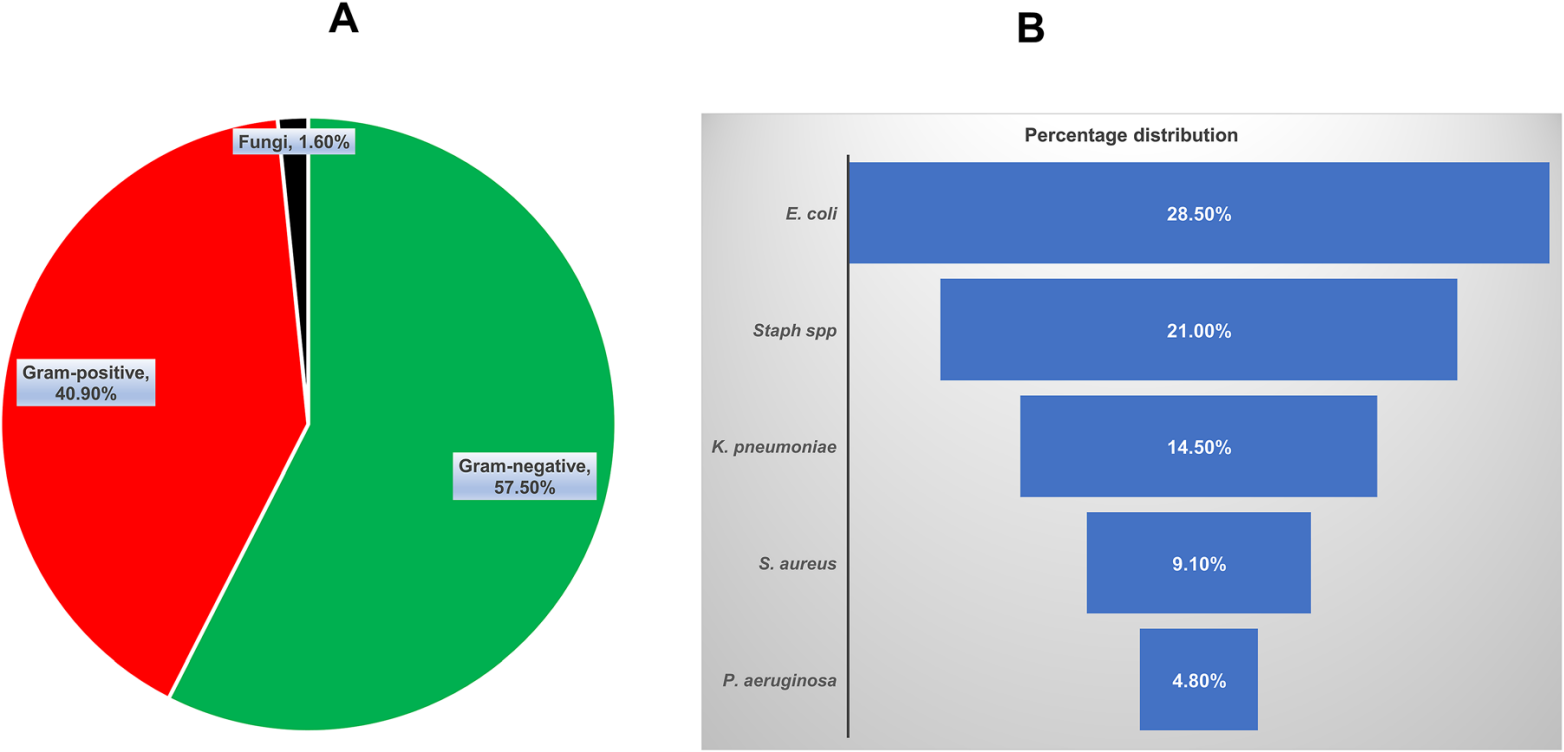
Pathogen distribution and composition. (A) Pie chart representation of the distribution and composition of the bacterial pathogens as identified by the separation gel coagulation tube combined with the MALDI-TOF MS. (B) A funnel demonstration of the top five pathogens identified by the separation gel coagulation tube combined with the MALDI-TOF MS.

A total of 186 bacterial strains were isolated from 186 positive blood culture specimens. Among them, 107 strains of Gram-negative bacteria accounted for 57.5 % (107/186); 76 strains of Gram-positive bacteria accounted for 40.9 % (76/186); and 3 strains of fungi accounted for 1.6 % (3/186), as shown in Figure 1A. The top five pathogens were *E. coli*, with 53 strains, accounting for 28.5 % (53/186); coagulase-negative *Staphylococcus* spp., accounting for 21.0 % (39/186); *Klebsiella pneumoniae*, accounting for 14.5 % (27/186); *Staphylococcus aureus*, accounting for 9.1 % (17/186) and *Pseudomonas aeruginosa*, accounting for 4.8 % (9/186) (Figure 1B).

### Direct identification results of separation gel coagulation tube pretreatment

#### Single-strain bloodstream infection

The identification rates of genus and species of 186 tested bacteria were 82.8 % (154/186) and 79.6 % (148/186), respectively. The identification rates of the top five pathogens at the species level were: *E. coli* 94.3 % (50/53), coagulase-negative *Staphylococcus* spp. 79.5 % (31/39), *K. pneumoniae* 92.6 % (25/27), *S. aureus* 82.4 % (14/17), and *P. aeruginosa* 88.9 % (8/9), data not shown.

#### Direct identification and assessment of the effectivity of the separation gel coagulation tube combined with MALDI-TOF MS compared to the conventional test method in Gram-negative bacteria identification

The identification rates of 107 strains of Gram-negative bacteria at the genus and species levels were 90.7 % (97/107) and 86.0 % (92/107), respectively, as shown in Table 1. Among them, the identification rates of *Enterobacteriales* spp. at the genus and species levels were 93.1 % (81/87) and 90.8 % (79/87), respectively. The identification rates of non-fermenting bacteria at the genus and species levels were 77.8 % (14/18) and 66.7 % (12/18), respectively; the identification rates of *Aeromonas* spp. at the genus and species levels were 100.0 % (2/2) and 50.0 % (1/2), respectively.

**Table 1:** Comparison of the Gram-negative bacteria identification results in the conventional test vs. the separation gel coagulation tube combined with the MALDI-TOF MS method in 186 positive blood culture bottles.

Organism	No of strains, n	Number (%) of isolates with correct identification to the genus level			Number (%) of isolates with correct identification to the species level		
		Conventional test	Separation gel coagulation tube combined with MALDI-TOF MS	p-Value	Conventional test	Separation gel coagulation tube combined with MALDI-TOF MS	p-Value
Gram-negative bacteria	107	92(86)	100(93.5)	0.07	92(86)	100(93.5)	0.07
Enterobacteriaceae	87	77(88.5)	79(90.8)	0.09	77(88.5)	79(90.8)	0.09
*Escherichia coli*	53	49(92.4)	50(94.3 %)	0.09	49(92.4)	50(94.3)	0.09
*Klebsiella pneumoniae*	27	24(88.8)	25(92.6)	0.088	24(88.8)	25(92.6)	0.088
*Enterobacter cloacae*	4	2(50)	2(50)	–	2(50)	2(50)	–
*Enterobacter aerogenes*	2	0(0.0)	1(50)	0.007	0(0.0)	1(50)	0.007
*Serratia marcescens*	1	1(100)	1(100)	–	1(100)	1(100)	–

We then compared the conventional bacteria method sensitivity to the combination of separation gel coagulation tube with MALDI-TOF MS in identifying Gram-negative bacteria to the genus and species level in the 186 positive culture bottles. According to our observations, there was no significant sensitivity difference between the conventional test and separation gel coagulation tube combined with MALDI-TOF MS in the Gram-negative bacteria, (92(86 %) vs. 100(93.5 %), p=0.07), *Enterobacteriaceae* spp*.*, 77(88.5 %) vs. 79(90.8 %), p=0.09, *E. coli,* 49(92.4 %) vs. 50(94.3 %), p=0.09, *Enterobacter cloacae* (2(50 %) vs. 2(50 %)), *K. pneumoniae* 24(88.8 %) vs. 25(92.6 %), p=0.088, and *Serratia marcescens.* However, there was a significant sensitivity difference between the use of the conventional test and the separation gel coagulation tube combined with MALDI-TOF MS in *Enterobacter aerogenes* identification, p=0.007, as shown in Table 1.

#### Direct identification and assessment of the efficiency of separation gel coagulation tube combined with MALDI-TOF MS compared to the conventional test approach in Gram-positive bacteria identification

The identification rates of 76 Gram-positive bacteria at the genus and species levels were 73.7 % (56/76) and 72.4 % (55/76), respectively, as shown in Table 2. The identification rates of *Staphylococcus* spp. at the genus and species levels were 80.4 % (45/56) and 75.0 % (42/56) respectively; the identification rates of *Streptococcus* spp. at the genus and species levels were 50.0 % (5/10) and 40.0 % (4/10), respectively; and the identification rates of *Enterococcus* spp. at the genus and species levels were 60.0 % (6/10) and 60.0 % (6/10), respectively.

**Table 2:** Comparison of the Gram-positive bacteria identification results in the conventional test vs. the separation gel coagulation tube combined with the MALDI-TOF MS method in 186 positive blood culture bottles.

Organism	No. of strains, n	Number (%) of isolates with correct identification to the genus level			Number (%) of isolates with correct identification to the species level		
		Conventional test	Separation gel coagulation tube combined with MALDI-TOF MS	p-Value	Conventional test	Separation gel coagulation tube combined with MALDI-TOF MS	p-Value
Gram-positive bacteria	76	35(46.0)	55(72.4)	0.003	35(46.0)	55(72.4)	0.003
*Staphylococcus* spp	56	39(69.6)	45(80.4)	0.001	39(69.6)	45(80.4)	0.001
*Staphylococcus epidermidis*	21	15(71.4)	17(81)	0.049	15(71.4)	17(81)	0.049
*Staphylococcus aureus*	17	10(58.8)	14(82.4)	0.002	10(58.8)	14(82.4)	0.002
*Staphylococcus hominis*	8	4(50)	6(75)	0.04	4(50)	6(75)	0.04
*Hemolytic Staphylococcus*	6	3(50)	5(83.3)	0.022	3(50)	5(83.3)	0.022
*Staphylococcus capitis*	4	3(75)	3(75)	–	3(75)	3(75)	–
*Enterococcus*	10	5(50)	6(60)	0.055	5(50)	6(60)	0.055
*Enterococcus faecalis*	6	4(66.7)	4(66.7)	–	4(66.7)	4(66.7)	–
*Enterococcus faecium*	4	1(25)	2(50)	0.043	1(25)	2(50)	0.043
*Streptococcus*	10	4(40)	7(70)	0.036	4(40)	7(70)	0.036
*Streptococcus mitis*	2	0(0.0)	0(0.0)	–	0(0.0)	(0.0)	–
*Streptococcus agalactiae*	2	0(0.0)	1(50)	0.002	0(0.0)	1(50)	0.002
*Streptococcus pneumoniae*	2	1(50)	1(50)	–	1(50)	1(50)	–
*Streptococcus sanguinis*	2	1(50)	1(50)	–	1(50)	1(50)	–
*Streptococcus dysgalactiae* subsp. *dysgalactiae*	2	0(0.0)	1(50)	–	0(0.0)	1(50)	–

In addition, we compared the sensitivity of the conventional bacteria method with the combination of separation gel coagulation tube with MALDI-TOF MS technique in identifying the Gram-positive bacteria to the genus and the species level in the 186 positive culture bottles. According to our results, there was a significant sensitivity difference between the conventional test and separation gel coagulation tube combined with MALDI-TOF MS in the Gram-positive bacteria, (35(46 %) vs. 55(72.4 %), p=0.003, *Staphylococcus* spp., 39(69.6 %) vs. 45(80.4 %), p=0.01, *Staphylococcus epidermidis*, 15(71.4 %) vs. 17(81 %), p=0.049, *S. aureus*, 10(58.8 %) vs. 14(82.4 %), p=0.002, *Staphylococcus hominis*, 3(50 %) vs. 6(75 %), p=0.04, hemolytic* Staphylococcus*, (3(50 %) vs. 5(83.3 %), p=0.022); *Enterococcus faecium,* 1(25 %) vs. 2(50 %), p=0.043); *Streptococcus* spp., 4(40 %) vs. 7(70 %), p=0.036); and *Streptococcus agalactiae*, 0(0.0 %) vs. 1(50 %), p=0.002), as shown in Table 5. However, we did not observe any significant sensitivity difference in the identification of *Staphylococcus capitis, Streptococcus mitis, Streptococcus pneumoniae, Streptococcus sanguinis, and Streptococcus dysgalactiae* subsp. *dysgalactiae,* as shown in Table 2.

#### Evaluation of the sensitivity of the separation gel coagulation tube combined with MALDI-TOF MS compared to the conventional test method in fungi identification

Finally, we compared the sensitivity of the conventional identification method with the combination of separation gel coagulation tube with MALDI-TOF MS in identifying fungi to the genus and species level in the 186 positive culture bottles. Our results confirmed a statistically significant sensitivity difference between the conventional method with the combination of separation gel coagulation tube with MALDI-TOF MS in the identification of fungi, and *Candida albicans*; 1(33.3 %) vs. 2(66.6 %), p=0.026, as shown in Table 3.

**Table 3:** Comparison of the fungi identification results in the conventional test vs. the separation gel coagulation tube combined with MALDI-TOF MS method in 186 positive blood culture bottles.

Organism	No. of strains, n	Number (%) of isolates with correct identification to the genus level			Number (%) of isolates with correct identification to the species level		
		Conventional test	Separation gel coagulation tube combined with MALDI-TOF MS	p-Value	Conventional test	Separation gel coagulation tube combined with MALDI-TOF MS	p-Value
Fungi	3	1(33.3)	2(66.6)	0.026	1(33.3)	2(66.6)	0.026
*Candida albicans*	3	1(33.3)	2(66.6)	0.026	1(33.3)	2(66.6)	0.026

#### The 4 and 6 h short-term solid-phase culture combined with MALDI-TOF MS in Gram-negative bacteria identification

We then studied the sensitivity of 4 h or 6 h short-term solid-phase culture combined with MALDI-TOF MS in identifying Gram-negative bacteria. We observed a compliance rate of 100.0 % for both 4 h or 6 h short-term solid-phase culture combined with MALDI-TOF MS in the identification of *E. coli*, *K. pneumoniae, E. cloacae, E. aerogenes,* and *P. aeruginosa.* However, we observed a 100 % compliance rate for a 6-h short-term solid phase culture combined with MALDI-TOF MS, but only a 50 % compliance rate for 4 h short-term solid phase culture combined with MALDI-TOF MS in the identification of *Acinetobacter baumannii, Stenotrophomonas maltophilia, Chryseobacterium indologenes* and *Aeromonas hydrophila*, as shown in Table 4.

**Table 4:** Comparison of short-term culture combined with MALDI-TOF MS results with conventional test results for Gram-negative bacteria specimens unidentified or identified to the genus level in 15 positive culture bottles.

Bacteria	Number, n	4 h short-term culture method			6 h short-term culture method		
		Combined with MALDI-TOF MS strains, %			Combined with MALDI-TOF MS strains, %		
		Biofilm formation (strain)	MALDI-TOF MS results (% of strains)	Compliance rate, %	Biofilm formation (strain)	MALDI-TOF MS Results (% of strains)	Compliance rate, %
*Escherichia coli*	3	3	3(100.0)	100.0	3	3(100.0)	100.0
*Klebsiella pneumoniae*	2	2	2(100.0)	100.0	2	2(100.0)	100.0
*Enterobacter cloacae*	2	2	2(100.0)	100.0	2	2(100.0)	100.0
*Enterobacter aerogenes*	1	1	1(100.0)	100.0	1	1(100.0)	100.0
*Pseudomonas aeruginosa*	1	1	1(100.0)	100.0	1	1(100.0)	100.0
*Acinetobacter baumannii*	2	1	1(50.0)	50.0	2	2(100.0)	100.0
*Stenotrophomonas maltophilia*	1	0	0(0.0)	0.0	1	1(100.0)	100.0
*Chryseobacterium indologenes*	2	0	0(0.0)	0.0	2	2(100.0)	100.0
*Aeromonas hydrophila*	1	1	1(100.0)	100.0	1	1(100.0)	100.0

#### The 4 and 6 h short-term solid phase culture combined with MALDI-TOF MS in Gram-positive bacteria and fungi identification

Finally, we studied the sensitivity of 4 h or 6 h short-term solid-phase culture combined with MALDI-TOF MS in the identification of Gram-positive bacteria and fungi. As per our results, the 6 h short-term solid phase culture combined with MALDI-TOF MS indicated 100.0 % compliance rate in the identification of *S. epidermidis, S. aureus, S. hominis, Hemolytic Staphylococcus, S. capitis, Enterococcus faecalis, S. agalactiae, S. pneumoniae*, and *E. faecium*, while the compliance rate for the identification of these microbes for the 4-h short-term solid phase culture combined with MALDI-TOF MS was 0.00 %. However, we did not observe any biofilm formation after a 4 or 6-h culture of *S. mitis, C. albicans*, and *S. sanguinis*, as depicted in Table 5.

**Table 5:** Comparison of short-term culture test combined with MALDI-TOF MS with conventional test results for Gram-positive bacteria and fungi specimens, unidentified or identified to the genus level in 22 positive culture bottles.

Bacteria	Number, n	4 h short-term culture method			6 h short-term culture method		
		Combined with MALDI-TOF MS strains, %			Combined with MALDI-TOF MS strains, %		
		Biofilm formation (strain)	MALDI-TOF MS results (% of strains)	Compliance rate, %	Biofilm formation (strain)	MALDI-TOF MS Results (% of strains)	Compliance rate, %
*Staphylococcus epidermidis*	4	0	0(0.0)	0.0	4	4(100.0)	100.0
*Staphylococcus aureus*	3	0	0(0.0)	0.0	3	3(100.0)	100.0
*Staphylococcus hominis*	2	0	0(0.0)	0.0	2	2(100.0)	100.0
*Hemolytic Staphylococcus*	1	0	0(0.0)	0.0	1	1(100.0)	100.0
*Staphylococcus capitis*	1	0	0(0.0)	0.0	1	1(100.0)	100.0
*Enterococcus faecalis*	2	0	0(0.0)	0.0	2	2(100.0)	100.0
*Enterococcus faecium*	2	0	0(0.0)	0.0	2	2(100.0)	100.0
*Streptococcus mitis*	2	0	0(0.0)	0.0	0	0(0.0)	0.0
*Streptococcus agalactiae*	1	0	0(0.0)	0.0	1	1(100.0)	100.0
*Streptococcus pneumoniae*	1	0	0(0.0)	0.0	1	1(100.0)	100.0
*Streptococcus sanguinis*	1	0	0(0.0)	0.0	0	0(0.0)	0(0.0)
*Candida albicans*	2	0	0(0.0)	0.0	0	0(0.0)	0(0.0)

Overall, for the positive blood culture specimens that were only identified to the genus level or not identified, we combined short-term solid-phase culture to improve the identification accuracy of this type of bacteria. The results showed that the MALDI-TOF MS identification accuracy within 4 h of short-term solid phase culture was 32.4 % (12/37), while the MALDI-TOF MS identification accuracy after 6 h of short-term solid phase culture was 86.5 % (32/37); except for *Streptococcus constellatus* subspecies, *S. sanguinis,* and *C. albicans*, which were not identifiable due to slow growth and no obvious biofilm. The remaining strains were identified to species level by MALDI-TOF MS combined with 6 h short-term culture, which was significantly higher than the detection rate of MALDI-TOF MS after 4 h short-term culture, and also confirmed that the enrichment method had little effect on bacterial activity.

## Discussion

The current study’s findings demonstrate the efficacy of MALDI-TOF MS combined with 4–6 h of short-term culture for the precise and rapid identification of pathogens in positive blood culture bottles. For example, among the 186 positive blood culture specimens analyzed, the combination of MALDI-TOF MS with a short-term culture of 4–6 h identified 57.5 % as Gram-negative, 40.9 % as Gram-positive bacteria, and 1.6 % as fungi. Our report agrees with the earlier findings that highlight the suitability of MALDI-TOF MS in detecting bacterial and fungal infections in blood specimens [10], 13], 14].

We also found that this method has higher accuracy in identifying Gram-negative bacteria compared to Gram-positive bacteria in positive blood culture bottles. Our observation is crucial since most bloodstream infections associated with high mortality have been traditionally attributed to Gram-negative bacteria [15], [16], [17]. While MALDI-TOF MS combined with a short-term culture of 4–6 h exhibited higher precision and accuracy in identifying Gram-negative bacteria, comparison with the conventional bacterial detection method revealed no significant differences, except for *E. aerogenes*. The findings are consistent with earlier studies that indicated the effectiveness of MALDI-TOF MS in detecting Gram-negative bacteria compared to Gram-positive bacteria [18], [19], [20].

The sensitivity test of 4 h or 6 h culture with MALDI-TOF MS in Gram-negative bacteria identification revealed a higher detection rate of MALDI-TOF MS in conjunction with 6-h compared to 4-h short-term culture for Gram-negative bacteria. Notably, Gram-negative bacteria identification yielded a 100 % compliance rate for MALDI-TOF MS when combined with a 6-h short-term culture, but the 4-h short-term culture compliance rate was 0.0 %. This observation may be attributed to the slower growth rate of Gram-positive bacteria relative to Gram-negative bacteria. These results suggest that a 6-h short-time culture coupled with MALDI-TOF MS is most effective for detecting the Gram-negative and Gram-positive bacterial infections in blood. Any bacterial detection method that provides accurate test results in a shorter time is most appropriate for bloodstream infections. The present study indicates that integrating MALDI-TOF MS with a short-term solid-phase culture method gives a significantly higher level of bacterial and fungal detection in positive blood samples within a reduced timeframe compared to the conventional identification method. This approach might be recommended in clinical laboratories to enhance the speed and accuracy of detecting bloodstream infections.

According to a previous study, the accuracy of MALDI-TOF MS in directly identifying pathogens in positive blood culture specimens depends on the pre-treatment method. Moreover, bacterial enrichment is key, and multiple pretreatment kits are commercially available [21]. The separation gel coagulation tube only contains gel and coagulant. After centrifugation, the gel can separate the bacteria from the broth. The spinning also separates blood cells from plasma. The experimental steps are simple, fast, and cost-effective, and pre-treatment can be improved according to the bacterial species after the enrichment of the bacteria to obtain better identification results [22]. The genus and species identification coincidence rates were 82.8 % (154/186) and 79.6 % (148/186), respectively. Among them, the genus and species identification coincidence rates of Gram-negative bacteria were 90.7 and 86.0 %, respectively; the genus and species identification coincidence rates of Gram-positive bacteria were 73.7 and 72.4 %, respectively, which are consistent with the previous findings [23], 24].

In this study, among 186 cases of single-strain bloodstream infection, the top five pathogens were *E. coli* (28.5 %), coagulase-negative *S. aureus* (21.0 %), *K. pneumoniae* (14.5 %), *S. aureus* (9.1 %), and *P. aeruginosa* (4.8 %). According to our findings, the separation gel coagulation tube method can achieve a species identification rate of 79.5–94.3 % for the mentioned pathogens. The results showed that this method accurately identifies these common bacteria. The identification agreement rates of *Enterococcus* spp., *Streptococcus* spp. and fungi were low, at 60.0, 40.0 and 33.3 %, respectively, which may be related to the following factors: insufficient mass spectrometry peaks obtained from these strains; high similarity of mass spectrometry peaks between different *Streptococcus* spp.; resistance of Gram-positive bacteria and fungi cell walls to dissolution; and interference from some residual blood proteins.

The results of the separation gel coagulation tube method combined with the short-term solid phase culture method in this study showed that among the 186 pathogens, 160 strains (86.0 %) could be accurately identified to the species level within 4 h; and 180 strains (96.8 %) could be accurately identified to the species level within 6 h. This finding shows that the combination of these two methods increase the bacterial detection rate and lead to accurate identification results. Compared with conventional identification methods, it significantly shortens the detection time. MALDI-TOF MS for rapid identification can substantially increase the proportion of patients receiving precision antibiotic treatment and provide clinicians with a timely basis for etiological diagnosis [25]. Rapid pathogen identification using MALDI-TOF MS enables earlier optimization of antimicrobial regimens, facilitates discontinuation of inappropriate anti-infective therapy, and significantly decreases the likelihood of treatment failure [26].

Although MALDI TOF has been reported as a rapid, simple, and reliable method for directly identifying pathogenic bacteria in blood culture bottles [20], this method has limitations. For instance, the MALDI-TOF MS identification rate is low during complex bacterial infection. Some researchers have increased the identification rate to 86.0 % by a short-term culture of the fluid containing two types of bacteria and then using MALDI-TOF MS for identification [27].

This study builds on the experience of the previous investigators, and there is no single standard procedure for the sample processing. Future research could include making more technical improvements to the pretreatment method of bacterial enrichment before direct identification by MALDI-TOF MS, and performing drug sensitivity tests directly on bacterial enrichments pretreated with separation gel coagulation tubes. Conducting rapid bacterial identification and drug sensitivity testing simultaneously can significantly reduce the time required to deliver results, enabling patients to receive timely and accurate antimicrobial treatment. This is particularly important, as MALDI-TOF requires less time than conventional methods for pathogen detection.

In general, our findings indicate that MALDI-TOF MS, combined with a 4–6-h short-term culture, enables rapid and precise direct pathogen identification from positive blood culture bottles. This approach achieved particularly high accuracy for Gram-negative bacteria, consistent with previous reports that MALDI-TOF MS demonstrates superior performance for Gram-negative organisms compared to Gram-positive organisms, due to differences in protein expression profiles and cell wall structure. These results reinforce earlier studies showing that short-term subculture before MALDI-TOF MS improves spectral quality and identification confidence [28], while also offering a clinically relevant turnaround time that can facilitate earlier antimicrobial optimization.

Our investigation had some limitations, since it only used data from a single institution. This study did not compare this combined method with other protocols for the direct identification of microorganisms from positive BC bottles. In addition, the number of bacterial isolates included in the present study was relatively low, and the interpretation was limited. Additionally, the limited sample size obtained in this study led to a restricted spectrum of bacterial species analyzed. Therefore, these results might not apply to other laboratories. Further investigation utilizing a larger number of more diverse samples over a longer period is thus warranted to validate these results. Future investigations may aim to combine this method with a rapid Antimicrobial Susceptibility Testing method and assess whether such identification with MALDI-TOF MS leads to better clinical outcomes. Finally, the success of the combination of separation gel coagulation tubes and MALDI-TOF MS demands meticulous preparation of samples, proper extraction protocols, and robust databases. To curb these challenges, a high level of vigilance must be ensured at every stage to ensure accurate and reproducible bacterial identification.

## Conclusions

In conclusion, a combination of separation gel coagulation tubes, short-term solid phase culture, and the MALDI-TOF MS direct identification of positive blood culture bottles using VITEK-MS is fast, effective, demonstrates high precision, and is feasible for routine testing. The Gram-negative bacteria identification is quicker and accurate compared to Gram-positive bacteria.
